# As Time Goes by: A rTMS Study on Age-Related Changes in Sentence Comprehension

**DOI:** 10.3389/fnagi.2018.00307

**Published:** 2018-10-30

**Authors:** Manuela Berlingeri, Desiré Carioti, Laura Danelli, Emanuele Lo Gerfo

**Affiliations:** ^1^DISTUM, Department of Humanistic Studies, University of Urbino Carlo Bo Urbino, Italy; ^2^NeuroMi, Milan Center for Neuroscience Milan, Italy; ^3^Center of Developmental Neuropsychology, ASUR Marche Pesaro, Italy; ^4^Department of Psychology, University of Milano-Bicocca Milan, Italy; ^5^Department of Economics, Management and Statistics, University of Milano-Bicocca Milan, Italy

**Keywords:** aging, sentence processing, compensatory processes, rTMS, precuneus, left inferior frontal gyrus, working memory

## Abstract

It is well established that off-line sentence judgment tasks (oSJTs) typically rely on phonological working memory (WM), beyond specific linguistic processing. Nevertheless, empirical findings suggest that a juvenile level of performance in an oSJT could be associated with the recruitment of age-specific additional supportive neural network in healthy aging. In particular, in one of our previous study, healthy elderlies showed the additional activation of associative visual cortices when compared with young controls. We suggested that age-related hyperactivations, during an auditory sentence judgment task, might represent the neurofunctional correlate of the recruitment of compensatory strategies that are necessary to maintain a juvenile level of performance. To explicitly test this hypothesis we adopted repetitive transcranial magnetic stimulation (rTMS). Twelve healthy elderlies and 12 young participants were engaged in an off-line semantic plausibility judgment task while rTMS was delivered over: (1) the left inferior frontal gyrus (LIFG; i.e., a core region of the WM network); (2) the precuneus; and (3) a Control Site (vertex). Results showed a significant main effect of Stimulation Site and a significant Group-by-Stimulation Site interaction effect. In particular, the rTMS stimulation of the LIFG slowed down reaction times (RTs) both in young and healthy elderly participants, while only healthy elderlies showed an increment of RTs during the stimulation of the precuneus. Taken together our results further support the idea that the maintenance of a juvenile level of performance in graceful aging may be associated with task-specific compensatory processes that would manifest them-selves, from the neurofunctional point of view, by the recruitment of additional neural supportive regions.

## Introduction

### Age-Related Changes in Working Memory Can Affect Sentence Comprehension

When one has to face with spoken messages the verbal information becomes available a bit at time and, therefore, temporally discontinuous inputs must be bound together to let the meaning of the entire message emerge. It is now well established that, beyond the analysis of phonological, lexical and syntactic information, sentence comprehension may depend, under some circumstances, also by the temporary storage of these representations in working memory (WM). In particular, while the role of WM may seem limited in on-line sentence processing (Caplan and Waters, 1999; Waters and Caplan, 2005; Caplan et al., 2011), the contribution of this temporary storage and processing system is critical for post-interpretative processes (i.e., processes that occur after the meaning of sentences has been extracted) that are typically called into cause during off-line sentence judgment tasks (oSJTs). According to the authors, post-interpretative processes would be based on a sort of phonological backup of the sentence that has to be interpreted (Caplan and Waters, 1999). Let focus on the sentence plausibility task, for example. In this case participants have to decide whether a sentence, for example “the planes fly in the sky,” is either plausible, or not, from the semantic point of view (i.e., whether it is true or false). Usually the plausibility of the sentence is revealed by the last word of the sentence it-self. As a consequence, to perform the task, subjects have to temporary store the entire sentence in their WM in order to express their judgment. Some behavioral studies suggest that, while the processing of plausible sentences (PSs; true sentences) may just be based on the activation of the semantic information conveyed by the sentence it-self, the processing of false sentences may involve additional cognitive steps as, in this last case, subjects have to solve a conflict between the sentence information stored in the WM and the semantic knowledge stored in long-term memory (Collins and Quillian, 1969; Glass and Holyoak, 1974). In the first case, the task resembles the typical recall of a trace from WM, while in the second case (i.e., with false sentences) participants may rely on additional reasoning, problem-solving or mental imagery skills.

This assumption, and the ensuing behavioral evidence (Caplan and Waters, 1999; Waters and Caplan, 2005), led the authors to explore the relationship between WM and post-interpretative processes *as time goes by*. Indeed, it is well established that WM capacity declines with age (Hedden and Gabrieli, 2004) and that this decline constrains cognition (and some aspect of language processing) in aging adulthood. According to the authors, the reduction of WM skills *as time goes by* would be related with the decrement of post-interpretative processes in off-line paradigms (Caplan and Waters, 1999). This reduction of efficiency would mainly manifest it-self with an increment of the reaction times to judge the sentences, on the one hand, and with a decrement of the level of judgment accuracy, at least in the case of the more complex stimuli, on the other hand (Obler et al., 1991; Waters and Caplan, 2005). Moreover, a structural equation modeling study showed that, while in on-line sentence judgments there are no effects of age and of WM, in oSJT there is both a direct effect of age, and an interaction between age and residual WM skills (DeDe et al., 2004).

Taken together the results reported in the literature suggest that the WM decline in aging may cause changes in the ability to interpret and judge spoken sentences (at least in off line situations). However, to date it is still unclear which one, among the different WM components, is mainly involved in post-interpretative processes and in their age-related changes. Part of the problem stems from the manipulation of syntactic complexity, and as a consequence of task-demand, in behavioral experiments; this, in turn, does not permit to distinguish between the contribution of the central executive and the contribution of the phonological loop (the two main cognitive candidates to the age-related decrement of off-line sentence comprehension skills).

To overcome this issue, we included in our study only syntactically simple sentences made by “subject + verb + complement”. By adopting such a simple (at least from the syntactic point of view) material, we were able to explore the neurocognitive dynamics associated with age-related changes in off-line sentence comprehension tasks.

### Neurofunctional Age-Related Changes in Sentence Comprehension

Sentence comprehension in healthy elderly is associated with a large-scale neural network involving two main dissociable components (Wingfield and Grossman, 2006): (i) the left peri-Sylvian language regions; and (ii) the left and right structures associated with the recruitment of WM and executive resources. In particular, the former component is associated with the activation of the ventral inferior frontal gyrus (vIFG), including Broca’s area, of the posterolateral temporal cortex, including Wernicke’s area, and of their interconnection (Caplan et al., 2000; Friederici, 2002; Luke et al., 2002); while the activation of the left dorsolateral prefrontal cortex (dlPFC), of the dorsal portion of left inferior frontal cortex (Smith et al., 1998; Chein and Fiez, 2001), bilaterally, and of the right posterolateral temporal cortex (Cooke et al., 2002) is associated with the latter component. Moreover, other areas could be activated during sentence comprehension when task-demand increases. For example, anterior cingulate activation was associated with increased attentional-demand during accelerated speech rate (Peelle et al., 2004), while subcortical regions, like the striatum, were activated during error monitoring when performing a difficult task (Wingfield and Grossman, 2006).

These widespread activations may support an accurate performance in sentence comprehension task in elderly subjects. In particular, executive resources could compensate the WM decline in healthy elderly during language tasks (Wingfield and Grossman, 2006), while language areas showed little age-related changes (Madden et al., 1996, 2002; Lustig and Buckner, 2004).

For example, a widespread pattern of activation in elderly compared to more focal activations in young adults emerged in the same language task (Cabeza, 2002; Logan et al., 2002).

To investigate the age-related changes both at the behavioral and the neurofunctional level, Grossman et al. (2002a) used a comprehension task in which sentences with a different burden for WM and at a different level of syntactic complexity were presented. Results showed that healthy elderlies hyperactivated some portions of the WM network to achieve a level of comprehension accuracy similar to the one of young controls. In a second study, Grossman et al. (2002b) divided the sample of healthy elderlies into two subgroups on the basis of behavioral performance: (a) good comprehenders, i.e., participants whose performance was similar to the young controls’ one; and (b) poor comprehenders, i.e., elderly participants with an impairment in sentences comprehension for particularly complex sentences. Interestingly, good comprehenders hyperactivated the dorsal portion of left inferior frontal cortex, a region that has been frequently associated with the WM (Paulesu et al., 1993; Smith et al., 1998; Chein and Fiez, 2001), and right posterior-lateral temporal-parietal regions, i.e., a pool of brain regions that was activated by young adults when challenged with complex sentences with a high WM-demand (Cooke et al., 2002).

On the contrary, Berlingeri et al. (2010a) showed that healthy elderlies that maintain, on average, a juvenile level of performance in an oSJT hyperactivated associative visual cortices and the medial temporal cortex when compared to young controls. This result had been interpreted as the neurofunctional manifestation of task-specific compensatory processes. In particular, the authors concluded that hyperactivations in healthy elderly might represent the adoption of *ad hoc* developed supportive strategies necessary to maintain a good behavioral performance; for example, the activation of associative visual cortices during an auditory oSJT may represent the recruitment of visual imagery strategies to support the age-related physiological decline of WM.

### Hypothesis and Aim

**Figure 1:**
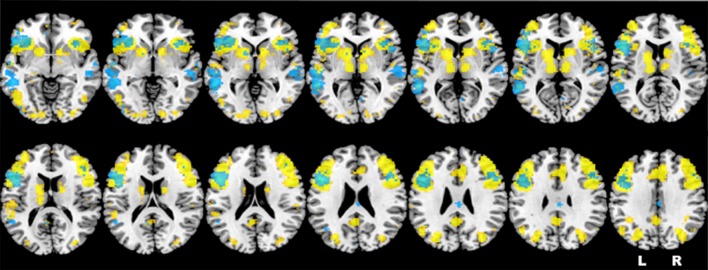
Representation of the neurofunctional overlap between sentence comprehension (in yellow) and verbal working memory (WM; in light blue), extracted by means of the Neurosynth toolbox. Brain regions highlighted in the figure are consistent with the activations identified by the first neuroimaging study about verbal WM (Paulesu et al., 1993) and by two recent meta-analyses (Wager and Smith, 2003; Rottschy et al., 2012).

Based on the results obtained in one of our previous studies (Berlingeri et al., 2010a) and on the literature about the neural correlates of sentence judgment in aging, we assume that healthy elderlies will be able to reach an adequate level of performance in an oSJT by adopting compensatory strategies to elude the age-related physiological WM decline. In this perspective, we assume that frontal areas related with WM process, and particularly the left IFG (LIFG), will be involved in oSJT together with other posterior brain areas related to imagery processes, as the precuneus, at least in elderly participants. Indeed, sentence comprehension is supported by a pool of brain regions that largely overlap to the neural network typically activated by verbal WM tasks, as shown also by the results of the automatic meta-analysis that we run by means of the Neurosynth Toolbox^1^ (see Figure 1).

According to this evidence, we expect to observe specific effects of LIFG stimulation both in young, both in elderly participants (as the LIFG is trivial for verbal WM tasks, and verbal WM is necessary to perform oSJT); on the contrary we assume that the inhibitory stimulation of the precuneus should affect only the group of elderlies to further support the compensatory hypothesis described also in the study by Berlingeri et al. (2010a)^2^.

To test these hypotheses, we implemented a repetitive transcranial magnetic stimulation (rTMS) paradigm: a group of healthy elderlies and a group of healthy young participants received an online inhibitory stimulation on: (i) LIFG; (ii) precuneus (P); and (iii) a Control Site (CZ), while they were performing an oSJT.

The reaction times (RTs) collected during the different stimulation conditions will allow us to understand the causal role of each single brain region in oSJT and to describe the ensuing age-related changes.

## Materials and Methods

### Participants

We recruited 12 healthy elderly participants and 12 young controls. However, we had to exclude two participants, one in each group^3^. As a result, we included in our analyses 11 neurologically healthy elderly (age 66.2 ± 5.27, range 21–31, years of educations 12.6 ± 2.61) and 11 young (age 23.4 ± 3.38, range 60–77, years of education 15.5 ± 0.93). All subjects were native Italian speakers, right-handed as indexed by the Edinburgh Handedness Inventory (Oldfield, 1971) and none had contraindications to receive TMS (Rossi et al., 2009). All the participants, with the exception of three young controls, were naïve to TMS stimulation. None of the participants had a history of neurological, psychiatric or neuropsychological disorders. None of the elderly volunteers had diabetes, hypertension or any other main medical condition.

Moreover, all the participants included in the group of healthy elderlies were assessed by means of a neuropsychological battery to exclude any cognitive deficit (see Table 1). The neuropsychological battery included tests of general cognitive level (MMSE; Magni et al., 1996; Folstein et al., 1975), abstract reasoning (Raven’s Progressive Matrices, Raven, 2000), long-term memory (Short Story Test, Carlesimo et al., 2002), verbal fluency (Novelli et al., 1986), visual-constructive skills (Rey’s Complex Figure, Carlesimo et al., 2002) and attentional abilities (Attentional Matrices, Spinnler and Tognoni, 1987; Trail Making Test A-B, Giovagnoli et al., 1996).

**Table 1:** Demographical variables of the sample and Mean (SD) performance (adjusted for age and education) obtained by elderlies participants in the neuropsychological assessment.

	Young group	Healthy elderly group		
Mean age (SD)	23.45 (3.38)	66.28 (5.27)		
Gender (M/F)	7/4	7/4		
Mean years of education (SD)	15.54 (0.93)	12.63 (2.61)		
**Cognitive function**		**Neuropsychological test**	**Mean (SD) scores**	**Cut-off**
Global measure		Mini Mental State Examination (MMSE, Magni et al., 1996; Folstein et al., 1975)	29.18 (1.16)	<23.8
Verbal long-term memory		Short Story test: immediate recall (Carlesimo et al., 2002)	5.52 (1.19)	<3.10
		Short Story test: delayed recall (Carlesimo et al., 2002)	4.74 (1.98)	<2.39
Visuo-spatial long-term memory		Rey’s Complex Figure: immediate recall (Carlesimo et al., 2002)	19.97 (5.13)	<6.44
		Rey’s Complex Figure: delayed recall (Carlesimo et al., 2002)	19.98 (4.89)	<6.33
Praxia		Rey’s Complex Figure: copy (Carlesimo et al., 2002)	34.68	<23.76
Problem solving		Raven’s Progressive Matrices (Raven, 2000)	32.27 (1.95)	<17.5
Attention		Trail making test (Giovagnoli et al., 1996)		
		Part A: numbers	52.2 (16.04)	≥94
		Part B: letters and numbers	105.5 (37)	≥283
		Part B–A	54.3 (28.43)	≥187
		Attentional matrices (Spinnler and Tognoni, 1987)	53.5 (6.45)	<30
Verbal fluency		Phonemic fluency (Novelli et al., 1986)	33.09 (9.08)	<16
		Semantic fluency (Spinnler and Tognoni, 1987)	23.31 (3.79)	<7

All subjects gave written informed consent in accordance with the Declaration of Helsinki. The protocol was approved by the “Ethical committee of the University of Milano-Bicocca” (General Assembly of the World Medical Association, 2014).

### Experimental Task

The task was conducted on a 17″ high-resolution PC computer screen using E-prime software 2.0 (Psychology Software Tools, Pittsburgh, PA, USA). Participants sat in front of the computer monitor in a semi-darkened room and were instructed to perform an off-line sentence judgment task: subjects were required to judge the semantic plausibility of the sentence presented in auditory modality as described in one of our previous study (Berlingeri et al., 2010a).

Each participant underwent three blocks of 48-sentence plausibility judgment that corresponded to the three stimulation sites described in the hypothesis section. The software randomly selected the presentation order of the sentences for each single participant, within each block. The order of the blocks was counterbalanced across participants.

**Figure 2:**
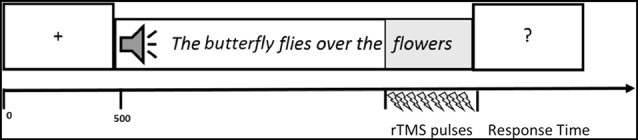
Graphic representation of one trial. A central fixation cross was presented for 500 ms at the beginning of each trial, then a sentence was auditorily administered over headphones to the participants. Immediately after the sentence presentation a question mark was showed in the center of the PC screen and participants were required to judge the sentence plausibility. During the listening of the last word of the sentence, repetitive transcranial magnetic stimulation (rTMS) was delivered.

A central fixation cross was presented for 500 ms at the beginning of each trial, then a sentence was auditorily administered over headphones to the participants. Immediately after the sentence presentation a question mark was showed in the center of the PC screen and participants were required to judge the sentence plausibility (see Figure 2). Plausibility judgment was made by pressing on the keyboard a green button for PSs and a red one for non-plausible sentences (NPSs) with the right and left forefinger. The position of the green and red buttons was counterbalanced across subjects. Judgment response time and accuracy were recorded. During the listening of the last word of the sentence, rTMS was delivered (see “TMS Procedure” section for more details).

Before each block, subjects underwent a training phase in which 12 sentences (not included in the set of the experimental stimuli) were delivered.

### Experimental Stimuli

A pool of 560 simple clauses (subject-verb-complement) was created (280 PSs, 280 NPSs). The NPSs were created by substituting the last content word with a new semantically unrelated word, without altering the syntactic structure of the entire clause. The old and the new last words were matched for number of syllables. The 560 sentences were administered to 60 college students that were asked to evaluate, on a five-steps Likert scale, the imageability and the level of “plausibility” of the sentences. As a result 72 highly imaginable PSs and 72 clearly implausible NPSs were selected. These sentences were divided into three lists that included 24 PSs and 24 NPSs each. The three lists were matched for: imageability, frequency and age of acquisition of the subject-word of the sentence (Barca et al., 2011), total number of syllables, imageability of the entire sentence and plausibility of the NPSs.

### Target Regions Identification

The target regions were identified on the basis of the neuroimaging data described in one of our previous articles (Berlingeri et al., 2010a). In particular, we run *ad hoc* second level analyses to test the differences between healthy elderlies and young participants in the sentence judgment task only.

We first isolated the LIFG and we tested the between-groups differences in this region only by means of a voxel-wise *t*-test. In particular, a significant reduction of activation was found in the LIFG (in a cluster located between *x* = −40, *y* = 24, *z* = −10 and *x* = −52, *y* = 28, *z* = 18^4^) in the group of healthy elderlies. The opposite comparison between healthy elderlies and young controls (namely the linear contrast “healthy elderlies > young controls”) returned a significant hyperactivation of higher order visual cortices, of the precuneus and of retrosplenial cortices bilaterally (in a cluster located between *x* = 10, *y* = −80, *z* = 50 and *x* = −8, *y* = −52, *z* = 8).

**Figure 3:**
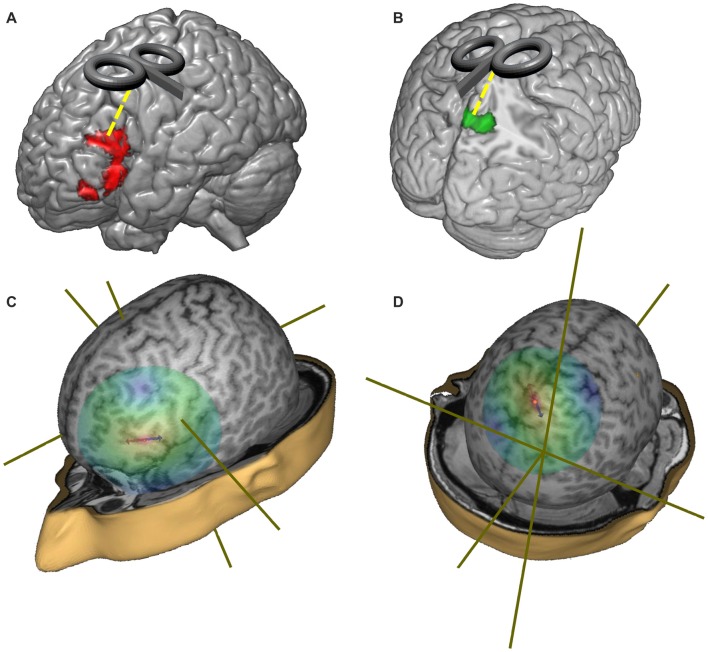
Representation of the target regions and of the distribution of the TMS magnetic field. **(A)** Red regions represent the hypo-activations in the off-line sentence judgment task (oSJT) in healthy elderlies (Berlingeri et al., 2010a). **(B)** Green regions represent elderlies’ specific activations during oSJT in the associative visual cortices (Berlingeri et al., 2010a). **(C)** Representation of the TMS magnetic field when the coil was centered over the left inferior frontal gyrus (LIFG) target region (MNI coordinate: *x* = −49; *y* = 21; *z* = 25). **(D)** Representation of the TMS magnetic field when the coil was centered over the precuneus target region (MNI: *x* = 0; *y* = −50; *z* = 65).

As a consequence, the stereotactic coordinate selected for the stimulation sites were: (i) *x* = −49, *y* = 21, *z* = 25 for the LIFG; (ii) *x* = 0, *y* = −50, *z* = 65 for the precuneus; while (iii) the vertex (Cz—MNI coordinate: *x* = 0; *y* = 0; *z* = 75) was used as a control region (see Figure 3). The Cz site is the most widely used control site for TMS studies because the auditory and somatosensory activations caused by vertex TMS can be equivalent to those of real TMS (Sandrini et al., 2011) and it is considered as a better control than other solutions (e.g., sham stimulation; Robertson et al., 2003).

### TMS Procedure

rTMS was administered with an Eximia™ TMS stimulator (Nexstim™, Helsinki, Finland) using a focal figure of eight 70-mm coil delivering biphasic pulse waveform. rTMS was delivered at 5-Hz frequency in trains of 1,400-ms duration (eight pulses) on the defined scalp site.

rTMS was applied at 110% of the rest motor threshold (rMT), the average rMT was 39.8% (range: 28%–50%) of the maximum stimulator output.

Before the experiment, each individual rMT was determined, following the up-dated guideline established by the International Federation of Clinical Neurophysiology, as the lowest intensity that produced motor evoked potentials of >50 μV peak-to-peak amplitude in at least five out of 10 trials with the muscles relaxed (Rossini et al., 2015). Electromyographic (EMG) traces were recorded from the left first dorsal interosseous (FDI) muscle using 9-mm diameter Ag–AgCl surface cup electrodes. The active electrode was placed over the left FDI and the reference electrode over the metacarpophalangeal joint of the index finger. The EMG signal was recorded using eXimia EMG (Nexstim™, Helsinki, Finland) amplifier, filtered with a band pass of 10–500 HZ and digitized at a sampling rate of 3 KHz.

Three cortical targets were identified in each subject on a high-resolution 3D volume (3D Magnetization Prepared Rapid Gradient-Echo) acquired on a 1.5T magnetic resonance scanner (Flip-angle = 20°, TE = 2.92 ms, TR = 9.16 ms, acquisition matrix: 256 × 256; interslice gap = 0 mm, and voxel size = 1 × 1 × 1 mm) using a navigated brain stimulation (NBS) system (Nexstim™, Helsinki, Finland), which employs a 3D infrared tracking position sensor unit, in order to map the position of the coil and participant’s head within the reference space of the individual’s MRI space. This allowed us to stimulate also a relatively “deep” brain region such as the precuneus although in its more external portion.

The coil was placed tangentially to the scalp, and adjusted for each participant in order to direct the electric field perpendicularly to the shape of the cortical gyrus, following the same procedure of a previous study (Mattavelli et al., 2013). Since TMS over LIFG site produced discomfort in one subject, the site of the stimulation was individually adjusted. Two of the elderlies did not complete the LIFG condition because of rTMS discomfort. For these participants we analyzed only the available trials.

The experiment was run in three blocks, one for each condition of stimulation (LIFG, P and Cz). The order of stimulation was counterbalanced across participants.

### Data Analysis

Statistical analyses were performed in the statistical programming environment R (R Development Core Team, 2008). For each single participant, only the RTs associated with correct responses were analyzed. Data were trimmed on the basis of the visual inspection of box-plots. In particular, we excluded all the RTs higher than 800 ms in the CZ stimulation condition, and all the RTs higher than 3,000 ms in the LIFG and P stimulation conditions.

As a first step, we run a series of step-wise General Linear Mixed effect Models (GLMM) using the LMER procedure available in the “lme4” R package (version 1.1-5, Bates et al., 2014). The model with the best fit to the data was selected on the basis of likelihood ratio test and goodness of fit indexes (Gelman and Hill, 2007). The results of this procedure are summarized in Table 2 (top panel).

**Table 2:** Likelihood ratio tests and goodness of fit indexes emerged by the Generalized Linear Mixed Models (GLMM).

	DF	AIC	BIC	logLik	Deviance	Chisq	DF_Chi_	Pr(>Chisq)
*RTs*								
M0	3	18890	18906	−9441.9	18884			
M1	4	18883	18904	−9437.5	18875	8.8866	1	0.002**
M2	6	18837	18868	−9412.5	18825	49.9989	2	0.000***
M3	8	18830	18872	−9407.0	18814	10.9230	2	0.000***
	**Accuracy levels**							
M0	2	102.01	106.36	−49.003	98.007			
M1	4	102.04	110.74	−47.020	94.040	3.9665	2	0.13
M2	5	102.67	113.55	−46.337	92.674	1.3662	1	0.24
M3	7	104.02	119.24	−45.011	90.021	2.6529	2	0.26

***p < 0.001; **p < 0.01; *p < 0.05.

Once the model was selected, we estimated effect sizes and we checked for residual distribution and GLM model assumptions by means of diagnostic plots. As the residuals were heteroscedastics and not normally distributed with a significant positive skewness, we opted for the application of generalized linear mixed model using the glmmPQL routine available in the *nlme library* (Pinheiro et al., 2018). The fixed effects were designed to test the main effect of Group, the main effect of Stimulation site and their interaction, to conform to the best Generalized Linear Mixed Model reported in Table 2 (top panel). The model was built using the following syntax:

Model<−glmmPQL(RT~Group*Stimulation site, ~1|Subject_ID, family=gaussian(link="log"),data=mydata,verbose=FALSE)

As clearly reported in the syntax, a by-subjects random intercept was included to account for participant-specific variability.

Lastly we explored performances of participants in term of accuracy, running a further Generalized Linear Mixed Model by means of the GLMER procedure. As in the case of RTs we designed a step-wise series of models with Group, Stimulation Site and their interaction as fixed effects. Also in this case we selected the model with the best fitting according to the likelihood ratio test and the goodness of fit indexes (Gelman and Hill, 2007), Table 2, bottom panel.

As clearly reported in Table 2 (bottom panel) none of the models was significant, but for sake of completeness we decided to report in the results section the effect of Group and of Stimulation Site according to the following syntax:

Model<−glmer(cbind(Correct_responses,Errors)~Stimulation Site+Group+(1+Subject_ID),data=mydata,family=binomial(link="logit"))

## Results

In what follows, we report the results of the GLMM run with the glmmPQL routine for RTs. Data analysis showed a significant main effect of Group ($X_{\left(1,1349\right)}^{2}$ = 10.36, *p* = 0.001), of the Stimulation Site ($X_{\left(2,1349\right)}^{2}$ = 86.69, *p* < 0.001) and a second-level Group-by-Stimulation Site interaction effect ($X_{\left(2,1349\right)}^{2}$ = 7.55, *p* = 0.02).

In particular, on average the RTs were higher when rTMS was delivered to the LIFG, rather than to the control site CZ, in both groups (CZ-LIFG_elderlies_: $X_{\left(1,1349\right)}^{2}$ = 51.07, *p*_FDR-corrected_ < 0.001; CZ-LIFG_young_: $X_{\left(1,1349\right)}^{2}$ = 10.44, *p*_FDR-corrected_ = 0.001).

**Figure 4:**
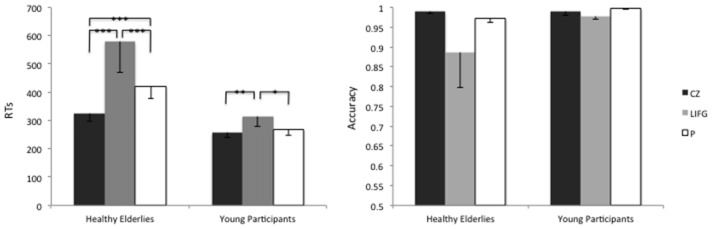
Average reaction times (RTs) and accuracy levels of young and healthy elderlies participants during the stimulation of the Control site (CZ), of the LIFG and of the precuneus (P). ***p < 0.001; **p < 0.01; *p < 0.05.

Furthermore, healthy elderly participants showed higher RTs than young controls during the stimulation of the LIFG (*t*_(1323)_ = 2.72; *p* = 0.006) and of the precuneus (*t*_(1323)_ = 1.97; *p* = 0.04), see Figure 4.

The accuracy level reached by healthy elderlies was similar to the one of young controls ($X_{\left(2,66\right)}^{2}$ = 1.37; *p* = 0.24), moreover, we did not find any effect of the Stimulation Site ($X_{\left(1,66\right)}^{2}$ = 4.08; *p* = 0.12).

## Discussion

In this study, we investigated the neurofunctional signatures of age-related changes underlying an oSJT. As described in the introduction, according to Caplan and Waters (1999) post-interpretative processes typically associated with oSJT entail the support of verbal WM to permit the phonological backup of the linguistic material that has to be judged.

Nevertheless, it is now well established that WM skills tend to decline as time goes by (Hedden and Gabrieli, 2004) even though language comprehension abilities, at least in not particularly demanding conditions, remain stable (Federmeier and Kutas, 2005; Thornton and Light, 2006). As suggested in one of our previous studies (Berlingeri et al., 2010a), this may be due to the fact that healthy elderlies are able to recruit additional and supportive neural networks that would represent the neurofunctional correlates of the activation of compensatory processes that are necessary to maintain a juvenile level of behavioral performance. In particular, according to our hypothesis, healthy elderlies would be able to adequately perform oSJT because of the activation of secondary visual cortices such as the middle occipital gyrus and the precuneus, i.e., of brain regions that are associated with visuo-spatial WM (Jonides et al., 1998; Zago et al., 2001; Mahayana et al., 2014) and with mental imagery (Fletcher et al., 1995; Cavanna and Trimble, 2006; Freton et al., 2014; Mashal et al., 2014). The causal relationship between the precuneus activation and the maintenance of a level of performance similar to the one of young controls in healthy elderlies challenged with an oSJT has been tested by means of rTMS. In what follows, we will discuss the role of our stimulation sites in oSJT to support the idea of a specific neurocognitive model of off-line sentence comprehension in aging.

### The Role of LIFG in Off-Line Sentence Processing Across Adult Life-Span

Sentence comprehension is supported by a pool of brain regions that largely overlap to the neural network typically activated by verbal WM tasks (see Figure 1).

This empirical evidence has largely contributed to the debate between those researchers that attribute a central role to verbal WM in sentence processing (Just and Carpenter, 1992; Papagno et al., 2007), and those supporting the idea that verbal WM would have a role in sentence processes only under specific experimental (task-related) conditions (Caplan et al., 2000; Waters and Caplan, 2001; Caplan et al., 2008). Notwithstanding this theoretical debate, the two positions convey in attributing a role of verbal WM in post-interpretative processes (Caplan and Waters, 1999) as compared to online sentence processing. In this kind of task indeed WM contributes to maintain the material that has to be either judged, or manipulated. Indeed, from the neurofunctional point of view, the higher cognitive demand associated with oSJT (as compared to online SJT) manifests it-self with the recruitment of a more complex neural network that includes also the anterior insula, SMA, and the inferior parietal cortex (Newman et al., 2009) beyond the classic perysilvian regions. In particular, the association between the PFC activity and post-interpretative process has been described in several neuroimaging studies (Caplan et al., 2000; Friederici, 2002; Luke et al., 2002; Wingfield and Grossman, 2006) that discuss this result within the WM framework. Moreover, the involvement of the LIFG in off-line sentence comprehension has been recently supported by the study of Giustolisi et al. (2018). Authors found that anodal tDCS delivered over the LIFG enhanced participants’ performances in an off-line sentence comprehension task regardeless the level of syntactic complexity of the linguistic material. This last result particularly fits with our findings that show a significant inhibitory effect of the rTMS over the LIFG, both in the elderlies, and in the young participants when required to judge a simple clause (with no additional syntactic manipulation). As previously described, the role of the LIFG in supporting syntactic processing has been largely debated (Friederici et al., 2003; Novick et al., 2005; Newman et al., 2009; Tyler et al., 2010), however, its involvement may be more easily associated with task specific conditions, rather than with a specific linguistic process. Accordingly, this brain region may be related with the processing of particularly challenging task-specific conditions (Vergallito et al., 2018), irrespectively by the fact that the task is requiring either a morpho-syntctic (Michael et al., 2001; Cooke et al., 2002) or a lexical processing (Keller et al., 2001; Xiao et al., 2005).

From a life-span point of view, WM deterioration in aging (Hedden and Gabrieli, 2004) seems to affect behavioral performances just in high demanding conditions, allowing elderlies to be effective in oSJT at least with less complex sentences. However, as observed by Grossman et al. (2002a) elderlies can perform at a juvenile level a sentence comprehension task even in particularly challenging conditions (i.e., with more complex sentences) by over-recruiting, on average, the LIFG and the right posterolateraltemporal-parietal junction.

Moreover, our results, fit well with the two-components model proposed by Wingfield and Grossman (2006). According to this model, healthy elderlies challenged with a sentence comprehension task would activate the *core sentence-processing network*, however, the maintenance of an adequate level of performance, also in more demanding conditions, would be supported by the additional rectuitment of the WM network and in particular of the more dorsal portion of the LIFG. Consequently, the maintenance of an adequate level of performance in healthy aging would be based on the activation of compensatory processes that would manifest them-selves with the additional recruitment of WM-related neural networks. Finally, the increment of RTs in the oSJT task recorded when the LIFG was stimulated in healthy elderlies (as compared to young controls) further supports the idea that this brain area has a fundamental role in sentence comprehension task and strengthens the neuroimaging results described by Wingfield and Grossman (2006): the direct comparison between good- and poor-comprehenders lets emerge a higher level of activation in a brain region that actually corresponded to our LIFG stimulation site.

### The “Compensatory” Role of the Precuneus in oSJT

According to the compensation hypothesis in healthy aging, the over-recruitment of additional brain regions during a specific cognitive task would represent the neurofunctional manifestation of the activation of the compensatory processes that are necessary to maintain a good level of behavioral performance (Cabeza, 2002).

Nevertheless some authors interpreted these findings from the opposite point of view: the “dedifferentiation hypothesis” (Li and Lindenberger, 1999). Accordingly, age-related hyperactivations would represent an unspecific neural response associated with the impossibility to efficiently engage the pool of task-specific brain regions. This neurofunctional phenomenon would manifest it-self with a progressive loss of cognitive specialization (Baltes and Lindenberger, 1997; Salthouse, 2001). However, according to Berlingeri et al. (2010a), there would be the chance of disentangling between these two hypotheses by simply looking at the behavioral performance. Indeed, compensatory process, by definition, should reflect the activation of an alternative supportive strategy to perform at an adequate level a specific task, on the contrary, a completely unspecific generalized activation should not be coupled, unless sporadically, with a behavioral success. In the study by Berlingeri et al. (2010a) both in the sentence judgment, both in the sentence recognition task, the elderlies’ performance was similar to the one of the young controls, but they showed hyperactivations of higher-order visual cortices (among these the precuneus) and of medial temporal regions that were interpreted in terms of compensatory processes.

To explicitly test the causal relationship between the activation of the precuneus and the maintenance of an adequate level of performance, in healthy elderlies, we used an rTMS suppression paradigm. The precuneus has been often associated with mental imagery, visuospatial attention and visual representation in the literature^5^ (Fletcher et al., 1995; Cavanna and Trimble, 2006; Freton et al., 2014; Mashal and Itkes, 2014; Utevsky et al., 2014; Bonnì et al., 2015). Interestingly, the inhibition caused by rTMS significantly affected only the RTs recorded in the groups of healthy elderlies, but not their level of accuracy. Taken together, results about time and accuracy allowed us to conclude, in line with our previous findings (Berlingeri et al., 2010a), that imagery and construction processes may represent an alternative strategy to support sentence rehearsal during post-interpretative processes in healthy elderlies. A more explicit experimental paradigm, based on the suppression of mental imagery (Dean et al., 2008) functions, for example, should be adopted to further support our hypothesis.

### Towards a Neurocognitive Model of Sentence Processing in Aging

To summarize, in our study the frontal TMS stimulation slowed down RTs both in young and elderly participants, confirming the key-role of LIFG (a core region of the WM network) in oSJT (Wingfield and Grossman, 2006) across the entire adulthood. On the contrary, only healthy elderlies were significantly affected by the stimulation of the precuneus. This result supports the hypothesis that high-level visual cortices, typically associated with imagery processes and with the construction of mental representations (Jackson et al., 2006; Jabbi et al., 2008; Hassabis and Maguire, 2007), can be called into cause during oSJT as time goes by.

With this regard, also the results concerning accuracy are noteworthy: both groups performed the task at the same level of accuracy, on average, but the slower RTs in elderlies support the idea that a more complex cognitive process was needed to reach this level of behavioral performance. Moreover, longer RTs in the group of elderly participants when the activity of the LIFG was inhibited is in line with the assumptions of the Compensation-Related Utilization of Neural Circuits Hypothesis (CRUNCH) model (Reuter-Lorenz and Cappell, 2008): healthy elderly would reach the saturation of the neurofunctional resources in the LIFG with a relatively easy task such as our oSJT, as a consequence, the slower RTs during the LIFG stimulation may represent the behavioral counterpart of the neurofunctional burden associated with this specific experimental condition. This in turn, would represent the *sine qua non* condition to activate compensatory processes that, in this specific case, would manifest them-selves with the activation of visuo-imaginative strategies supported by the activity of higher visual cortices. As a final remark, we would like to suggest that the results of this study could set the rationale to develop new interventional programs based on non-invasive brain stimulation techniques, such as TMS and tDCS (see Cespón et al., 2018 for a recent review and Cespón et al., 2017 for a recent empirical protocol of intervention). Indeed, in one of our previous study (Berlingeri et al., 2010b) we showed that MCI patients were unable to recruit the secondary visual cortices during oSJT, while kept on relying over the recruitment of the LIFG. This sort of “neurofunctional inflexibility” was coupled with a significant behavioral impairment at the task of interest to further suggest that pathological aging might be characterized by the impossibility to spontaneously recruit compensatory strategies. In the light of this evidence, it would be interesting to test, in future studies, whether an *ad hoc* created interventional program may reduce the “neurofunctional inflexibility” of MCI patients by triggering the recruitment of compensatory networks.

## Author Contributions

MB and ELG contributed to the conception and design of the study. LD and DC collected behavioral data. ELG administered rTMS. MB and DC performed the statistical analysis. MB wrote the first draft of the manuscript. DC, LD and ELG wrote sections of the manuscript. All authors contributed to manuscript revision, read and approved the submitted version.

## Conflict of Interest Statement

The authors declare that the research was conducted in the absence of any commercial or financial relationships that could be construed as a potential conflict of interest.

## Footnotes

^1^http://neurosynth.org

^2^To make our hypothesis clearer, at least from the methodological point of view, we are interested in exploring the group-by-stimulation site interaction effect.

^3^One of the healthy elderly was excluded due to a technical problem during the TMS stimulation. A young control was excluded because of a technical problem during the recording of RTs.

^4^The stereotactic coordinates are reported according to the Montreal Neurological Institute (MNI) standardized space.

^5^The posterior brain coordinates that we selected is associated with cognitive processes as imagination (posterior probability = 0.88; Jackson et al., 2006; Jabbi et al., 2008; Decety et al., 2013), construction of mental representations (posterior probability = 0.89; Hassabis and Maguire, 2007), autobiographical memory (posterior probability = 0.86; Maguire et al., 2010; Wilbers et al., 2012); and episodic memory (posterior probability = 0.79; Suzuki et al., 2009; Irish et al., 2014; Oertel-Knöchel et al., 2015), as emerged by automated meta-analysis run through the Neurosynth toolbox (http://neurosynth.org/.

## References

[B1] Baltes P. B. Lindenberger U. (1997). Emergence of a powerful connection between sensory and cognitive functions across the adult life span: a new window to the study of cognitive aging? Psychol. Aging 12, 12–21. 10.1037//0882-7974.12.1.12 9100264

[B2] Barca L. Burani C. Arduino L. S. (2011). Lexical and Sublexical Variables: Norms for 626 Italian Nouns. Available online at: http://www.istc.cnr.it/it/grouppage/lexvar.

[B4] Bates D. Maechler M. Bolker B. Walker S. (2014). lme4: linear mixed-effects models using Eigen and S4. R Package Vers. 1, 1–23.

[B5] Berlingeri M. Bottini G. Danelli L. Ferri F. Traficante D. Sacheli L.. (2010a). With time on our side? Task-dependent compensatory processes in graceful aging. Exp. Brain Res. 205, 307–324. 10.1007/s00221-010-2363-7 20680252

[B6] Berlingeri M. Sacheli L. Danelli L. Ferri F. Traficante D. Basilico S.. (2010b). Neurofunctional and neuromorphological evidence of the lack of compensation in pathological aging. Behav. Neurol. 23, 185–187. 10.3233/BEN-2010-0290 21422550 PMC5434398

[B7] Bonnì S. Koch G. Miniussi C. Bassi M. S. Caltagirone C. Gainotti G. (2015). Role of the anterior temporal lobes in semantic representations: paradoxical results of a cTBS study. Neuropsychologia 76, 163–169. 10.1016/j.neuropsychologia.2014.11.002 25445777

[B8] Cabeza R. (2002). Hemispheric asymmetry reduction in older adults: the HAROLD model. Psychol. Aging 17, 85–100. 10.1037//0882-7974.17.1.85 11931290

[B12] Caplan D. Alpert N. Waters G. Olivieri A. (2000). Activation of Broca’s area by syntactic processing under conditions of concurrent articulation. Hum. Brain Mapp. 9, 65–71. 10.1002/(sici)1097-0193(200002)9:2<65::aid-hbm1>3.0.co;2-4 10680763 PMC6871836

[B10] Caplan D. Chen E. Waters G. (2008). Task-dependent and task-independent neurovascular responses to syntactic processing. Cortex 44, 257–275. 10.1016/j.cortex.2006.06.005 18387556 PMC2427191

[B11] Caplan D. Dede G. Waters G. Michaud J. Tripodis Y. (2011). Effects of age, speed of processing, and working memory on comprehension of sentences with relative clauses. Psychol. Aging 26, 439–450. 10.1037/a0021837 21480714

[B9] Caplan D. Waters G. S. (1999). Verbal working memory and sentence comprehension. Behav. Brain Sci. 22, 77–94; discussion 95–126. 10.1017/S0140525X99001788 11301522

[B13] Carlesimo G. A. Buccione I. Fadda L. Graceffa A. Mauri M. Lorusso S. (2002). Standardizzazione di due test di memoria per uso clinico: breve racconto e figura di rey. Nuova Rivista Neurol. 12, 1–13.

[B14] Cavanna A. E. Trimble M. R. (2006). The precuneus: a review of its functional anatomy and behavioural correlates. Brain 129, 564–583. 10.1093/brain/awl004 16399806

[B15] Cespón J. Miniussi C. Pellicciari M. C. (2018). Interventional programmes to improve cognition during healthy and pathological ageing: cortical modulations and evidence for brain plasticity. Ageing Res. Rev. 43, 81–98. 10.1016/j.arr.2018.03.001 29522820

[B16] Cespón J. Rodella C. Rossini P. M. Miniussi C. Pellicciari M. C. (2017). Anodal transcranial direct current stimulation promotes frontal compensatory mechanisms in healthy elderly subjects. Front. Aging Neurosci. 9:420. 10.3389/fnagi.2017.00420 29326582 PMC5741680

[B17] Chein J. M. Fiez J. A. (2001). Dissociation of verbal working memory system components using a delayed serial recall task. Cereb. Cortex 11, 1003–1014. 10.1093/cercor/11.11.1003 11590110

[B18] Collins A. M. Quillian M. R. (1969). Retrieval time from semantic memory. J. Verbal Learn. Verbal Behav. 8, 240–247. 10.1016/s0022-5371(69)80069-1

[B19] Cooke A. Zurif E. B. DeVita C. Alsop D. Koenig P. Detre J.. (2002). Neural basis for sentence comprehension: grammatical and short-term memory components. Hum. Brain Mapp. 15, 80–94. 10.1002/hbm.10006 11835600 PMC6872024

[B20] Dean G. M. Dewhurst S. A. Whittaker A. (2008). Dynamic visual noise interferes with storage in visual working memory. Exp. Psychol. 55, 283–289. 10.1027/1618-3169.55.4.283 18683625

[B21] Decety J. Chen C. Harenski C. Kiehl K. A. (2013). An fMRI study of affective perspective taking in individuals with psychopathy: imagining another in pain does not evoke empathy. Front. Hum. Neurosci. 7:489. 10.3389/fnhum.2013.00489 24093010 PMC3782696

[B22] DeDe G. Caplan D. Kemtes K. Waters G. (2004). The relationship between age, verbal working memory and language comprehension. Psychol. Aging 19, 601–616. 10.1037/0882-7974.19.4.601 15584786

[B23] Federmeier K. D. Kutas M. (2005). Aging in context: age-related changes in context use during language comprehension. Psychophysiology 42, 133–141. 10.1111/j.1469-8986.2005.00274.x 15787850

[B24] Fletcher P. C. Frith C. D. Baker S. C. Shallice T. Frackowiak R. S. Dolan R. J. (1995). The mind’s eye—precuneus activation in memory-related imagery. Neuroimage 2, 195–200. 10.1006/nimg.1995.1025 9343602

[B25] Folstein M. F. Folstein S. E. McHugh P. R. (1975). “Mini-mental state”. A practical method for grading the cognitive state of patients for the clinician. J. Psychiatr. Res. 12, 189–198. 10.1016/0022-3956(75)90026-6 1202204

[B26] Freton M. Lemogne C. Bergouignan L. Delaveau P. Lehericy S. Fossati P. (2014). The eye of the self: precuneus volume and visual perspective during autobiographical memory retrieval. Brain Struct. Funct. 219, 959–968. 10.1007/s00429-013-0546-2 23553546

[B27] Friederici A. D. (2002). Towards a neural basis of auditory sentence processing. Trends Cogn. Sci. 6, 78–84. 10.1016/s1364-6613(00)01839-8 15866191

[B28] Friederici A. D. Rueschemeyer S.-A. Hahne A. Fiebach C. J. (2003). The role of left inferior frontal and superior temporal cortex in sentence comprehension: localizing syntactic and semantic processes. Cereb. Cortex 13, 170–177. 10.1093/cercor/13.2.170 12507948

[B29] Gelman A. Hill J. (2007). Data Analysis Using Regression and Multilevelhierarchical Models. New York, NY: Cambridge University Press.

[B103] General Assembly of the World Medical Association (2014). World Medical Association Declaration of Helsinki: ethical principles for medical research involving human subjects. J. Am. Coll. Dent. 81, 14–18. 25951678

[B30] Giovagnoli A. R. Del Pesce M. Mascheroni S. Simoncelli M. Laiacona M. Capitani E. (1996). Trail making test: normative values from 287 normal adult controls. Ital. J. Neurol. Sci. 17, 305–309. 10.1007/bf01997792 8915764

[B31] Giustolisi B. Vergallito A. Cecchetto C. Varoli E. Romero Lauro L. J. (2018). Anodal transcranial direct current stimulation over left inferior frontal gyrus enhances sentence comprehension. Brain Lang. 176, 36–41. 10.1016/j.bandl.2017.11.001 29175380

[B32] Glass A. L. Holyoak K. (1974). The effect ofsome andall on reaction time for semantic decisions. Mem. Cognit. 2, 436–440. 10.3758/bf03196901 21274770

[B33] Grossman M. Cooke A. DeVita C. Alsop D. Detre J. Chen W.. (2002a). Age-related changes in working memory during sentence comprehension: an fMRI study. Neuroimage 15, 302–317. 10.1006/nimg.2001.0971 11798267

[B34] Grossman M. Cooke A. DeVita C. Chen W. Moore P. Detre J.. (2002b). Sentence processing strategies in healthy seniors with poor comprehension: an fMRI study. Brain Lang. 80, 296–313. 10.1006/brln.2001.2581 11896643

[B35] Hassabis D. Maguire E. A. (2007). Deconstructing episodic memory with construction. Trends Cogn. Sci. 11, 299–306. 10.1016/j.tics.2007.05.001 17548229

[B36] Hedden T. Gabrieli J. D. (2004). Insights into the ageing mind: a view from cognitive neuroscience. Nat. Rev. Neurosci. 5, 87–96. 10.1038/nrn1323 14735112

[B37] Irish M. Piguet O. Hodges J. R. Hornberger M. (2014). Common and unique gray matter correlates of episodic memory dysfunction in frontotemporal dementia and Alzheimer’s disease. Hum. Brain Mapp. 35, 1422–1435. 10.1002/hbm.22263 23670951 PMC6869668

[B38] Jabbi M. Bastiaansen J. Keysers C. (2008). A common anterior insula representation of disgust observation, experience and imagination shows divergent functional connectivity pathways. PLoS One 3:e2939. 10.1371/journal.pone.0002939 18698355 PMC2491556

[B39] Jackson P. L. Brunet E. Meltzoff A. N. Decety J. (2006). Empathy examined through the neural mechanisms involved in imagining how I feel versus how you feel pain. Neuropsychologia 44, 752–761. 10.1016/j.neuropsychologia.2005.07.015 16140345

[B40] Jonides J. Schumacher E. H. Smith E. E. Koeppe R. A. Awh E. Reuter-Lorenz P. A.. (1998). The role of parietal cortex in verbal working memory. J. Neurosci. 18, 5026–5034. 10.1523/JNEUROSCI.18-13-05026.1998 9634568 PMC6792554

[B41] Just M. A. Carpenter P. A. (1992). A capacity theory of comprehension: individual differences in working memory. Psychol. Rev. 99, 122–149. 10.1037/0033-295x.99.1.122 1546114

[B42] Keller T. A. Carpenter P. A. Just M. A. (2001). The neural bases of sentence comprehension: a fMRI examination of syntactic and lexical processing. Cereb. Cortex 11, 223–237. 10.1093/cercor/11.3.223 11230094

[B43] Li S.-C. Lindenberger U. (1999). “Cross-level unification: a computational exploration of the link between deterioration of neurotransmitter systems and dedifferentiation of cognitive abilities in old age,” in Cognitive Neuroscience of Memory, Hogrefe and Huber, eds Nilsson L.-G. Markowitsch H. J. (Kirkland, WA: Hogrefe & Huber), 103–146.

[B44] Logan J. M. Sanders A. L. Snyder A. Z. Morris J. C. Buckner R. L. (2002). Under-recruitment and nonselective recruitment: dissociable neural mechanisms associated with aging. Neuron 33, 827–840. 10.1016/S0896-6273(02)00612-8 11879658

[B45] Luke K. K. Liu H. L. Wai Y. Y. Wan Y. L. Tan L. H. (2002). Functional anatomy of syntactic and semantic processing in language comprehension. Hum. Brain Mapp. 16, 133–145. 10.1002/hbm.10029 12112767 PMC6871887

[B46] Lustig C. Buckner R. L. (2004). Preserved neural correlates of priming in old age and dementia. Neuron 42, 865–875. 10.1016/j.neuron.2004.04.002 15182724

[B47] Madden D. J. Langley L. K. Denny L. L. Turkington T. G. Provenzale J. M. Hawk T. C.. (2002). Adult age differences in visual word identification: functional neuroanatomy by positron emission tomography. Brain Cogn. 49, 297–321. 10.1006/brcg.2001.1502 12139956 PMC1810390

[B48] Madden D. J. Turkington T. G. Coleman R. E. Provenzale J. M. DeGrado T. R. Hoffman J. M. (1996). Adult age differences in regional cerebral blood flow during visual world identification: evidence from H215O PET. Neuroimage 3, 127–142. 10.1006/nimg.1996.0015 9345484

[B490] Magni E. Binetti G. Bianchetti A. Rozzini R. Trabucchi M. (1996). Mini-mental state examination: a normative study in Italian elderly population. Eur. J. Neurol. 3, 198–202. 10.1111/j.1468-1331.1996.tb00423.x 21284770

[B49] Maguire E. A. Kumaran D. Hassabis D. Kopelman M. D. (2010). Autobiographical memory in semantic dementia: a longitudinal fMRI study. Neuropsychologia 48, 123–136. 10.1016/j.neuropsychologia.2009.08.020 19720072 PMC2806951

[B50] Mahayana I. T. Tcheang L. Chen C.-Y. Juan C.-H. Muggleton N. G. (2014). The precuneus and visuospatial attention in near and far space: a transcranial magnetic stimulation study. Brain Stimul. 7, 673–679. 10.1016/j.brs.2014.06.012 25112521

[B51] Mashal N. Itkes O. (2014). The effects of emotional valence on hemispheric processing of metaphoric word pairs. Laterality 19, 511–521. 10.1080/1357650x.2013.862539 24328525

[B52] Mashal N. Vishne T. Laor N. (2014). The role of the precuneus in metaphor comprehension: evidence from an fMRI study in people with schizophrenia and healthy participants. Front. Hum. Neurosci. 8:818. 10.3389/fnhum.2014.00818 25360101 PMC4199320

[B53] Mattavelli G. Rosanova M. Casali A. G. Papagno C. Romero Lauro L. J. (2013). Top-down interference and cortical responsiveness in face processing: a TMS-EEG study. Neuroimage 76, 24–32. 10.1016/j.neuroimage.2013.03.020 23523809

[B54] Michael E. B. Keller T. A. Carpenter P. A. Just M. A. (2001). fMRI investigation of sentence comprehension by eye and by ear: modality fingerprints on cognitive processes. Hum. Brain Mapp. 13, 239–252. 10.1002/hbm.1036 11410952 PMC6872122

[B55] Newman S. D. Lee D. Ratliff K. L. (2009). Off-line sentence processing: what is involved in answering a comprehension probe? Hum. Brain Mapp. 30, 2499–2511. 10.1002/hbm.20684 19184993 PMC6870822

[B56] Novelli G. Papagno C. Capitani E. Laiacona M. (1986). Tre test clinici di ricerca e produzione lessicale. Taratura su sogetti normali. Arch. Psicol. Neurol. Psichiatr. 47, 477–506.

[B57] Novick J. M. Trueswell J. C. Thompson-Schill S. L. (2005). Cognitive control and parsing: reexamining the role of Broca’s area in sentence comprehension. Cogn. Affect. Behav. Neurosci. 5, 263–281. 10.3758/cabn.5.3.263 16396089

[B58] Obler L. K. Fein D. Nicholas M. Albert M. L. (1991). Auditory comprehension and aging: decline in syntactic processing. Appl. Psycholinguist. 12, 433–452. 10.1017/s0142716400005865

[B59] Oertel-Knöchel V. Reinke B. Matura S. Prvulovic D. Linden D. E. van de Ven V. (2015). Functional connectivity pattern during rest within the episodic memory network in association with episodic memory performance in bipolar disorder. Psychiatry Res. 231, 141–150. 10.1016/j.pscychresns.2014.11.014 25575881

[B60] Oldfield R. C. (1971). The assessment and analysis of handedness: the Edinburgh inventory. Neuropsychologia 9, 97–113. 10.1016/0028-3932(71)90067-4 5146491

[B61] Papagno C. Cecchetto C. Reati F. Bello L. (2007). Processing of syntactically complex sentences relies on verbal short-term memory: evidence from a short-term memory patient. Cogn. Neuropsychol. 24, 292–311. 10.1080/02643290701211928 18416493

[B62] Paulesu E. Frith C. D. Frackowiak R. S. (1993). The neural correlates of the verbal component of working memory. Nature 362, 342–345. 10.1038/362342a0 8455719

[B63] Peelle J. E. McMillan C. Moore P. Grossman M. Wingfield A. (2004). Dissociable patterns of brain activity during comprehension of rapid and syntactically complex speech: evidence from fMRI. Brain Lang. 91, 315–325. 10.1016/j.bandl.2004.05.007 15533557

[B64] Pinheiro J. Bates D. DebRoy S. Sarkar D. R Core Team (2018). nlme: Linear and Nonlinear Mixed Effects Models. R package version 3.1–131.1. Available online at: https://CRAN.R-project.org/package=nlme

[B102] R Development Core Team (2008). R: A Language and Environment for Statistical Computing. Vienna: R Foundation for Statistical Computing.

[B65] Raven J. (2000). The Raven’s progressive matrices: change and stability over culture and time. Cogn. Psychol. 41, 1–48. 10.1006/cogp.1999.0735 10945921

[B66] Reuter-Lorenz P. A. Cappell K. A. (2008). Neurocognitive aging and the compensation hypothesis. Curr. Dir. Psychol. Sci. 17, 177–182. 10.1111/j.1467-8721.2008.00570.x

[B67] Robertson E. M. Theoret H. Pascual-Leone A. (2003). Studies in cognition: the problems solved and created by transcranial magnetic stimulation. J. Cogn. Neurosci. 15, 948–960. 10.1162/089892903770007344 14614806

[B68] Rossi S. Hallett M. Rossini P. M. Pascual-Leone A. Safety of TMS Consensus Group. (2009). Safety, ethical considerations, and application guidelines for the use of transcranial magnetic stimulation in clinical practice and research. Clin. Neurophysiol. 120, 2008–2039. 10.1016/j.clinph.2009.08.016 19833552 PMC3260536

[B69] Rossini P. M. Burke D. Chen R. Cohen L. Daskalakis Z. Di Iorio R.. (2015). Non-invasive electrical and magnetic stimulation of the brain, spinal cord, roots and peripheral nerves: basic principles and procedures for routine clinical and research application. An updated report from an IFCN Committee. Clin. Neurophysiol. 126, 1071–1107. 10.1016/j.clinph.2015.02.001 25797650 PMC6350257

[B70] Rottschy C. Langner R. Dogan I. Reetz K. Laird A. R. Schulz J. B.. (2012). Modelling neural correlates of working memory: a coordinate-based meta-analysis. Neuroimage 60, 830–846. 10.1016/j.neuroimage.2011.11.050 22178808 PMC3288533

[B71] Salthouse T. A. (2001). Attempted decomposition of age-related influences on two tests of reasoning. Psychol. Aging 16, 251–263. 10.1037/0882-7974.16.2.251 11405313

[B72] Sandrini M. Umilta C. Rusconi E. (2011). The use of transcranial magnetic stimulation in cognitive neuroscience: a new synthesis of methodological issues. Neurosci. Biobehav. Rev. 35, 516–536. 10.1016/j.neubiorev.2010.06.005 20599555

[B73] Smith E. E. Jonides J. Marshuetz C. Koeppe R. A. (1998). Components of verbal working memory: evidence from neuroimaging. Proc. Natl. Acad. Sci. U S A 95, 876–882. 10.1073/pnas.95.3.876 9448254 PMC33811

[B74] Spinnler H. Tognoni G. (1987). Italian group on the neuropsychological study of ageing: italian standardization and classification of neuropsychological tests. Ital. J. Neurol. Sci. 6, 1–120. 3330072

[B75] Suzuki C. Tsukiura T. Mochizuki-Kawai H. Shigemune Y. Iijima T. (2009). Prefrontal and medial temporal contributions to episodic memory-based reasoning. Neurosci. Res. 63, 177–183. 10.1016/j.neures.2008.11.010 19110014

[B76] Thornton R. Light L. L. (2006). “Language comprehension and production in normal aging,” in Handbook of the Psychology of Aging (Sixth Edition), eds Birren J. E. Schaie K. W. (Amsterdam: Elsevier), 261–287.

[B77] Tyler L. K. Wright P. Randall B. Marslen-Wilson W. D. Stamatakis E. A. (2010). Reorganization of syntactic processing following left-hemisphere brain damage: does right-hemisphere activity preserve function? Brain 133, 3396–3408. 10.1093/brain/awq262 20870779 PMC2965424

[B78] Utevsky A. V. Smith D. V. Huettel S. A. (2014). Precuneus is a functional core of the default-mode network. J. Neurosci. 34, 932–940. 10.1523/JNEUROSCI.4227-13.2014 24431451 PMC3891968

[B79] Vergallito A. Romero Lauro L. J. Bonandrini R. Zapparoli L. Danelli L. Berlingeri M. (2018). What is difficult for you can be easy for me Effects of increasing individual task demand on prefrontal lateralization: a tDCS study. Neuropsychologia 109, 283–294. 10.1016/j.neuropsychologia.2017.12.038 29288683

[B80] Wager T. D. Smith E. E. (2003). Neuroimaging studies of working memory. Cogn. Affect. Behav. Neurosci. 3, 255–274. 10.3758/cabn.3.4.255 15040547

[B82] Waters G. S. Caplan D. (2001). Age, working memory, and on-line syntactic processing in sentence comprehension. Psychol. Aging 16, 128–144. 10.1037/0882-7974.16.1.128 11302362

[B81] Waters G. S. Caplan D. (2005). The relationship between age, processing speed, working memory capacity, and language comprehension. Memory 13, 403–413. 10.1080/09658210344000459 15952262

[B83] Wilbers L. Deuker L. Fell J. Axmacher N. (2012). Are autobiographical memories inherently social? Evidence from an fMRI study. PLoS One 7:e45089. 10.1371/journal.pone.0045089 23028774 PMC3448611

[B84] Wingfield A. Grossman M. (2006). Language and the aging brain: patterns of neural compensation revealed by functional brain imaging. J. Neurophysiol. 96, 2830–2839. 10.1152/jn.00628.2006 17110737

[B85] Xiao Z. Zhang J. X. Wang X. Wu R. Hu X. Weng X.. (2005). Differential activity in left inferior frontal gyrus for pseudowords and real words: an event-related fMRI study on auditory lexical decision. Hum. Brain Mapp. 25, 212–221. 10.1002/hbm.20105 15846769 PMC6871751

[B86] Zago L. Pesenti M. Mellet E. Crivello F. Mazoyer B. Tzourio-Mazoyer N. (2001). Neural correlates of simple and complex mental calculation. Neuroimage 13, 314–327. 10.1006/nimg.2000.0697 11162272

